# GrainGenes: Tools and Content to Assist Breeders Improving Oat Quality

**DOI:** 10.3390/foods11070914

**Published:** 2022-03-23

**Authors:** Victoria C. Blake, Charlene P. Wight, Eric Yao, Taner Z. Sen

**Affiliations:** 1 Department of Plant Sciences and Plant Pathology, Montana State University, Bozeman, MT 59717, USA; victoria.blake@montana.edu; 2 Western Regional Research Center, Crop Improvement and Genetics Research Unit, United States Department of Agriculture—Agricultural Research Service, Albany, CA 94710, USA; ericiam@berkeley.edu; 3 Ottawa Research and Development Centre, Agriculture and Agri-Food Canada, Ottawa, ON K1A 0C6, Canada; charlene.wight@agr.gc.ca; 4 Department of Bioengineering, University of California, Berkeley, CA 94720, USA

**Keywords:** oat, quality, test weight, protein content, fatty acids, beta-glucan, genome browser, comparative mapping

## Abstract

GrainGenes is the USDA-ARS database and Web resource for wheat, barley, oat, rye, and their relatives. As a community Web hub and database for small grains, GrainGenes strives to provide resources for researchers, students, and plant breeders to improve traits such as quality, yield, and disease resistance. Quantitative trait loci (QTL), genes, and genetic maps for quality attributes in GrainGenes represent the historical approach to mapping genes for groat percentage, test weight, protein, fat, and β-glucan content in oat (*Avena* spp.). Genetic maps are viewable in CMap, the comparative mapping tool that enables researchers to take advantage of highly populated consensus maps to increase the marker density around their genes-of-interest. GrainGenes hosts over 50 genome browsers and is launching an effort for community curation, including the manually curated tracks with beta-glucan QTL and significant markers found via GWAS and cloned cellulose synthase-like AsClF6 alleles.

## 1. Introduction

Cultivated oat (*Avena sativa* L.) has been recognized as a valuable food source for millennia. As a cereal, oats provide beneficial levels of carbohydrates, such as starch and soluble dietary fiber, as well as lipids, protein, and several B vitamins [1]. Postharvest, the primary attributes of oat grain quality include test weight, groat percentage, and lipid, protein, and β-glucan content. Understanding and mapping the underlying genes for these traits has long been a goal of small grains research.

The potential for quality in oat grain is primarily due to the genetic makeup of the varieties selected for production. Oat breeders strive to achieve excellent grain quality while maintaining high yield and disease resistance. Phenotypic characterization of germplasm is the cornerstone of this process, but many traits, particularly those with polygenic inheritance, are not easy nor quick to measure. Because of this, molecular markers have been developed throughout the last several decades to assist breeders in the selection of elite germplasm early in the breeding process (e.g., marker-assisted selection). Databases became necessary to organize the enormous amount of information and remain a stable resource for crop research groups.

GrainGenes (https://wheat.pw.usda.gov, accessed on 17 March 2022) has been a comprehensive database for published data relating to molecular markers, elite germplasm, genetic maps, and now, genome browsers for the small grains wheat, barley, rye, oat, and their relatives, since 1992 [2,3]. GrainGenes is designed and funded to ensure long-term data sustainability for small grains researchers. Additionally, serving as a community Web hub, GrainGenes provides a permanent home for raw data such as nursery reports, annual reports, gene catalogs, job announcements and funding opportunities, a calendar, etc. The importance of centralized databases, such as GrainGenes, lies in the following fact: when related datasets are distributed without any linking between them, such as in the Supplementary Materials sections of disparate manuscripts, the knowledge that can be obtained from these datasets is limited. Bringing them together in a centralized location, having them linked by expert curators, and allowing them to be visualized through appropriate online tools enables researchers to access information more easily and create a greater knowledge base.

In this paper, we show both the curated data content and the tools to access and visualize it, so that researchers can assess the value of the linked datasets and harness them for their own research.

In collaboration with colleagues from both private and public institutions, GrainGenes recently added a wealth of legacy and current *Avena* spp. markers, genes, quantitative trait loci (QTL), germplasm, and genetic maps. GrainGenes also hosts two hexaploid *A. sativa* oat browsers (OT3098 PepsiCo 2020, 2021; https://wheat.pw.usda.gov/jb?data=/ggds/oat-ot3098v2-pepsico, accessed on 17 March 2022) and two diploid oat browsers from A. atlantica and A. eriantha [4]. Several more browsers are in the production stage and may well be available by the time of publication. 

QTL define regions of the genome responsible for quantitative phenotypic traits such as height, oil content, and degree of pathogen resistance. Identifying markers and chromosome map regions containing QTL is typically the first step in identifying the precise gene (s) responsible for a phenotype. Over time, markers have evolved from being based on simple phenotypic characters, to using radioactive labor-intensive techniques, to using fast and inexpensive sequence-based approaches. While bi-parental mapping populations segregating for specific traits were most often used in the past [5,6,7], more recent studies often employ the use of association mapping panels comprising hundreds of different lines from different genetic backgrounds [8,9,10]. The CMap application [11] in GrainGenes provides a tool for the comparison of these datasets, thus enriching the marker density around genes in sparsely populated maps by virtue of common markers.

At the time of this writing, GrainGenes contains records for 392 *Avena* spp. genes in 87 gene classes (Table 1), 1126 QTL in 60 trait categories (Table 2), and 1683 *Avena* genetic maps in 160 “mapdata” sets. The ‘Genetic Maps at GrainGenes’ link from the project homepage goes to a complete and interactive list of all genetic maps, and Table 3 provides a summary of the ten most recent *Avena* spp. genetic map sets published from 2018–2020. Using all of the information and tools comprising the GrainGenes database, the oat research community can now rapidly identify regions of the oat genome to target for crop improvement, including those affecting grain quality.

## 2. How to Search and Reach Information on GrainGenes

**Figure 1:**
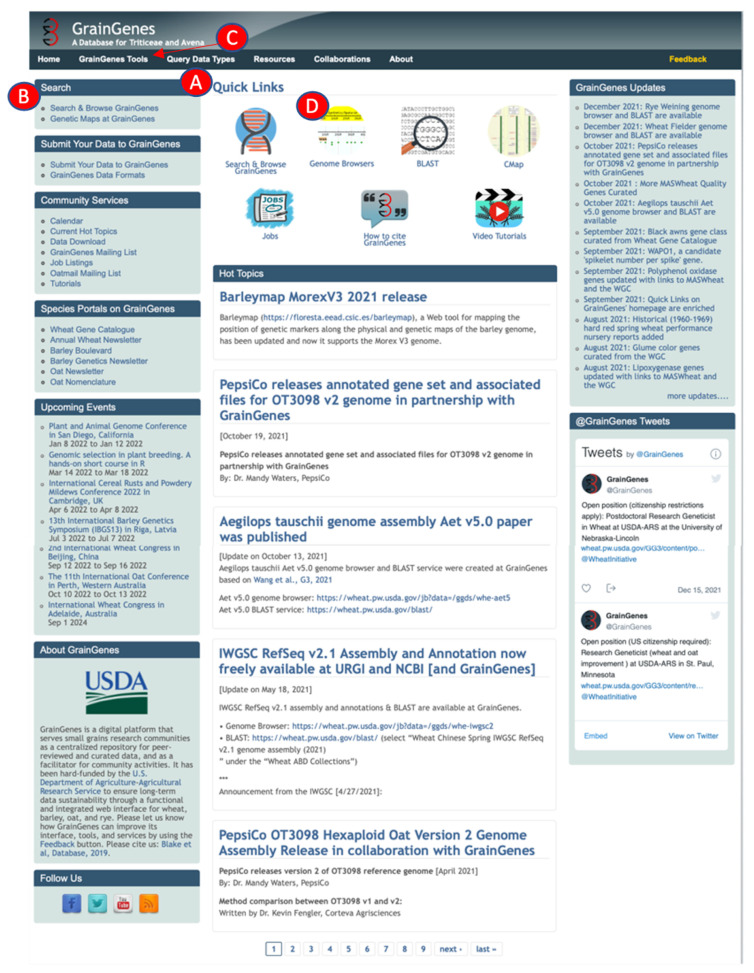
The GrainGenes homepage in January 2022. Highlighted features are (**A**) Quick Links for the most popular features; (**B**) One of three ‘Search and Browse’ portals; (**C**) Menu for advanced search tools; and (**D**) Genome Browser collection.

Accessing information about oat quality traits and oat genome browsers in GrainGenes provides researchers with rich information content across a wide range of data types. Figure 1 is a screenshot of the GrainGenes home page (https://wheat.pw.usda.gov, accessed on 17 March 2022) with just a few portals described.

(A). The Quick Links section in the middle of GrainGenes’ home page provides an intuitive and visually informative landing area that links out to various pages containing useful tools and resources for users.
(B). “Search and Browse GrainGenes” within the Quick Link collection, on the left menu, and in the GrainGenes Tools dropdown on the top menu, leads to a page that contains three different types of search capabilities for static and dynamic pages (https://wheat.pw.usda.gov/cgi-bin/GG3/browse.cgi, accessed on 17 March 2022), as well as external datasets related to wheat through the Wheat Information System (WheatIS) [12]. Since the third search type is specific to wheat, here we will only discuss the first two search types: (1) for static pages, users can search pre-indexed GrainGenes Web and database pages by using the search algorithm provided by Google™; (2) for dynamic pages within the GrainGenes database, which includes a wide range of data types such as genetic markers, genetic maps, QTL, traits, and phenotypes, users are able to deploy flexible search terms encompassing either all types of data or a specific data class. Users can browse a specific data type by clicking on the data class on the search page.
(C). Within the GrainGenes Tools dropdown menu, “Advanced Queries” allows advanced users to access data programmatically using structured query language (SQL). Data models and database schema are provided here. For those who are interested in writing their own codes to access data in GrainGenes, we encourage them to visit the site at https://wheat.pw.usda.gov/GG3/advanced-queries, accessed on 17 March 2022 and follow the links there. Batch queries within the GrainGenes Advanced Query menu enable users to query more than one object; for example, to list all the QTL, significant markers, and maps for a given trait. As with the SQL Interface page, pre-made queries are also provided as an example.
(D). A direct link into the collection of genome browsers. All individual browsers are grouped by crop, and colorful links take the user to the selected browser or open another group of browsers, in the case of pangenomes. The top collection is the most current or most popular browser for each crop. At the time of this writing, the most current genome browser for *Avena* is the PepsiCo OT3098 v2 Hexaploid oat (2021).

## 3. Oat Grain Quality Information in the GrainGenes Database

Genetic maps of genes and QTL relating to the oat grain quality traits mentioned below are represented as interactive online maps in GrainGenes, and are available to the CMap utility for visualization, comparison to other maps, and marker enrichment around genes of interest using common markers with densely populated consensus maps [13]. Populating the GrainGenes database for oat data is an ongoing project in collaboration with colleagues at Agriculture and Agri-Food Canada (AAFC) with expertise in oat. All oat data added to GrainGenes since 2018 were ‘pre-curated’ at AAFC, providing simple addition to GrainGenes and resulting in a comprehensive and current collection of oat maps and QTL.

### 3.1. Test Weight

**Figure 2:**
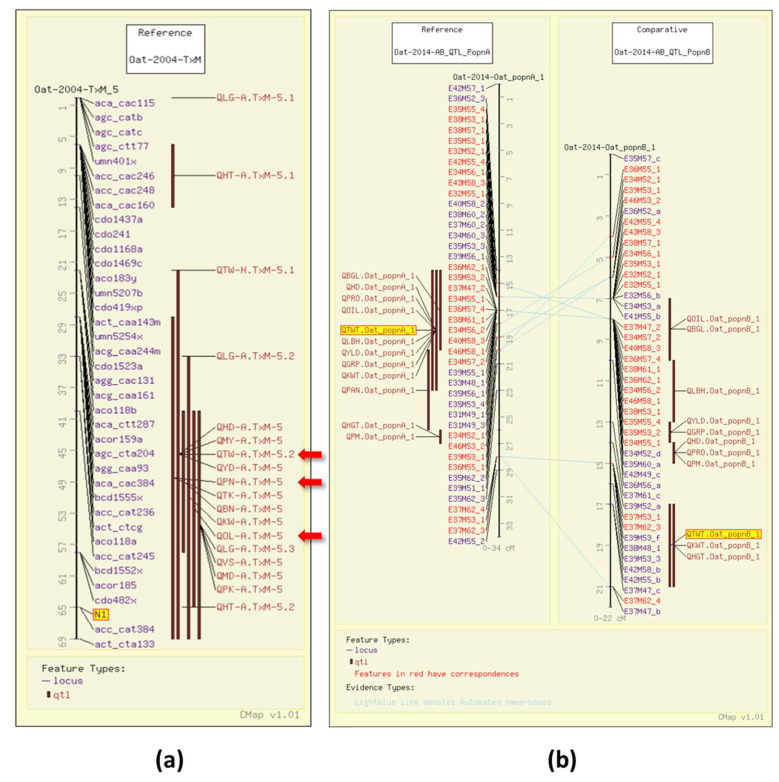
Test weight (TWT) QTL illustrated on the CMap map viewing/comparative mapping tool. (**a**) Several QTL for quality and agronomic traits overlapping with the N1 locus (highlighted) for the covered/hulless trait on the Terra x Marion population. QTL also discussed below are highlighted with red arrows, these include QTW-A.TxM-5.2 for test weight, QPN-A.TxM-5 for protein content, and QOL-A.TxM-5 for groat oil content.; (**b**) Alignment of linkage groups from two advanced backcross Iltis populations illustrating overlapping QTL within populations including test weight QTL (highlighted). Note the QTL map to the same linkage groups, as determined by the number of common markers, but map to different regions.

Test weight (TWT), or bulk density, is a measure of weight per unit volume of grain. Test weight is the result of the groat/grain ratio, with a high groat/grain size ratio indicating a lack of air in the space between the hull and the kernel. It is an indicator of milling quality in oat [14]. In 2004, four TWT QTL were identified in a Terra x Marion (TxM) population (GrainGenes Mapdata: Oat-2004-TxM) [6]. One of the test weight QTL in the TxM cross was associated with the gene for the covered/hulless trait (N1) segregating in that population (Figure 2a). In 2014, QTL were mapped in two Iltis advanced backcross populations with amplified fragment length polymorphism (AFLP) markers (GrainGenes Mapdata: Oat-2014-AB_QTL). [15]. The comparative mapping tool, CMap, in GrainGenes [11] enables researchers to easily compare genetic maps, similar to the ones resulting from this study (Figure 2b). 

As sequencing technologies have become more affordable, it is now feasible for many labs to map QTL for traits using a method called a genome-wide association study (GWAS) [8,9,10]. This method uses a large collection of markers to quickly scan a large number of subjects. In human studies, this may involve scanning the genomes of several thousand unrelated patients for a particular gene variant associated with a disease. In plants, we use collections of germplasm that have been collected worldwide (i.e., landraces), curated collections of elite cultivars from breeding programs, or mixtures of both. By measuring traits in these collections and testing the DNA from all of the members, researchers can associate regions of the genome with the wide number of traits found in a large, varied collection, making this a very efficient way to map QTL. 

GWAS on the Collaborative Oat Research Enterprise (CORE) collection of elite cultivars by Esvelt Klos et al. [8] mapped three mean test weight (TWT) QTL, one QTL for TWT variance, and one QTL associated with both TWT variance and mean across location years with known map locations. QTL with significant markers that were not mapped on the most recent consensus map for oat [13] include three for TWT variance, one QTL that maps with mean groat content, and one that maps with groat content variance. They also found five QTL that influenced TWT (including a QTL for kernel weight and groat content variance) to be additive, as the mean test weight increased with the presence of one to five QTL among the 343 lines evaluated. These data and the other GWAS QTL from this paper will be curated into GrainGenes records in early 2022. 

The GrainGenes database has the DNA sequences of hundreds of thousands of molecular markers, many of which were found to be significant for traits of interest, but were not genetically mapped in the original studies. Oat researchers have discovered thousands of single nucleotide polymorphism (SNP) markers, which means that one specific nucleotide varies within a DNA span of, for example, 100 base pairs (bp) among different cultivars. Overall, 100 bp is actually a very long query sequence when carrying out a BLAST search [16,17]. A new feature in GrainGenes is the ability to BLAST any DNA sequence onto any of over 150 BLAST databases. Fifty-five of these are assembled genomes present in GrainGenes as individual JBrowse genome browsers. This will enable a confident chromosome assignment within the QTL record in the GrainGenes database, and the creation of a private genome browser track with the QTL aligned to the DNA sequence on any oat genome browser by virtue of the BLAST sequence alignment of the significant marker and the pseudomolecule (DNA sequence).

### 3.2. Groat Percentage

Groat percentage (also sometimes described as “milling yield”, “hull content”, or “groat content (GC)”) measures the proportion of dehulled whole groats relative to the entire grain, and is another major indicator of milling quality. Groat percentage is under the control of one main gene and three modifying genes, but some influence of the environment is also observed [5]. As with most polygenic traits, however, the precise transcripts controlling this trait have not yet been identified. In 2001, groat percentage was mapped in the populations Kanota x Ogle and Kanota x Marion and two QTL from two different regions were mapped in each of them (GrainGenes Mapdata: Oat-2001-KxO-QTL and Oat-2001-KxM-QTL [18]. Several traits were mapped in this study. A QTL for kernel length was reported in both KxO and KxM near one of the GC QTL in each population, and a QTL for kernel width was reported in KxO at the same genetic location as a QTL for groat percentage, suggesting that a change in kernel size or shape could have an influence on groat percentage.

In the Iltis advanced backcross populations [15], one QTL maps to the same linkage group in both populations (see Figure 2b; QGRP.Oat_popnA_1 and QGRP.Oat_popnB_1), and an additional QTL is mapped in Population “B” (GrainGenes Mapdata: Oat-2014-AB_QTL_PopnA, Oat-2014-AB_QTL_PopnB). As with test weight (TWT), QTL for the mean and variance of “groat content (GC)” were mapped by GWAS in the CORE Collection [8]. Two QTL for GC variance, one for GC mean and variance, and one multi-trait QTL for GC mean, plump mean and variance, and thin kernels mean, are described across location years with known genetic map locations for significant markers. Four QTL for GC variance are described, one that shares a significant marker with mean TWT and one QTL for controlling both mean TWT and mean GC whose significant markers are unmapped.

### 3.3. Grain Protein

Cereal grains store proteins in their endosperm to nourish the developing plant embryo during germination and are an important nutrient for human and animal food. Storage proteins called prolamins are found only in the grasses, and those in oats are called ‘avenins’. While prolamins from wheat (gliadins) and barley (hordeins) are known to be toxic to those with the autoimmune disorder celiac disease, the safety of avenins in the diets of celiac patients is still under review [19]. Avenins in oat grains account for 10–20% of the storage proteins, the remaining ones being globulins [20]. New uses for oat protein have been described, including as a plant-derived gelling agent [21], and in other food processing technologies [22].

The quality of oat protein due to a high content of the essential amino acid lysine has been known for decades, and now is known to be from the globulin fraction [23]. In 1978, the first efficient extraction and purification of avenin was described and a common genetic ancestor to prolamins in the Triticeae is suggested, due to the similarity in amino acid composition [24]. Characterization of grain storage proteins reflects the history of molecular markers in modern biology. The first avenin markers mapped were based on gel electrophoresis patterns of the proteins themselves [25]. In 1990, genomic clones were described that represent the two major classes of storage proteins, an avenin and a 12 S globulin [26]. Avenin gene clones [27] and oat globulin gene clones [28] were later developed into Restriction Fragment Length Polymorphism (RFLP] markers and Sequence Characterized Amplified Region (SCAR) markers [29], which could then be used to identify specific genomic regions carrying these genes in later studies. These markers are not just important storage protein loci, but also serve as markers for clusters of rust resistance genes, as first reported by Howes et al. [30]. This serves as an important example of how genetic markers can be used to track other genes of interest by virtue of their genetic linkage, and not by common causal genes for the traits. 

As more uses are found for oat protein, a breeding target will naturally be grain with higher quantities of protein in the endosperm and/or variation in the quality of the proteins. Although the gene for a specific protein may have been cloned, the expression of that gene, the stability of the transcript, etc. are still under the control of several other genetic factors and these QTL are a target to develop high-protein oats.

**Figure 3:**
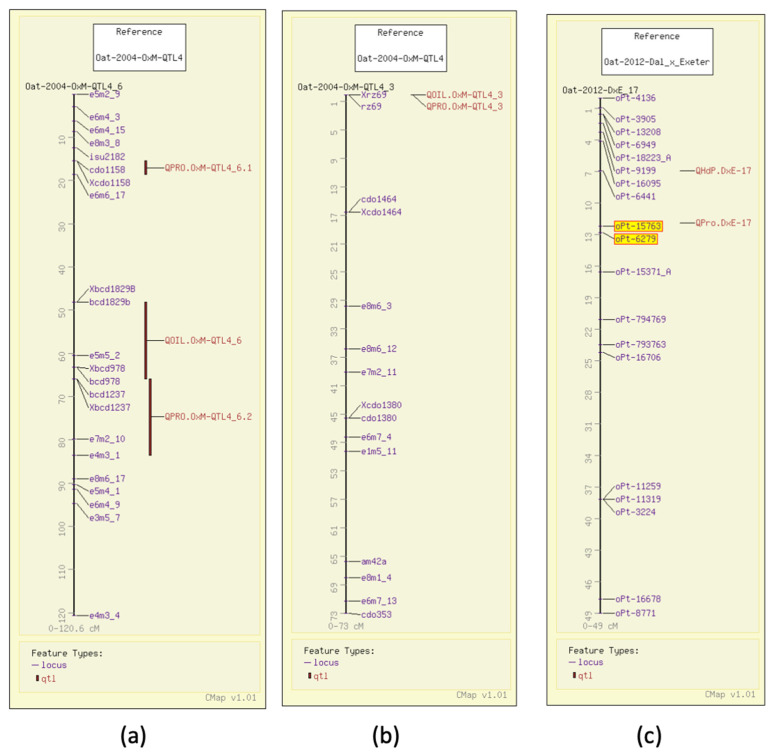
Grain protein QTL illustrated on the CMap map viewing/comparative mapping tool. (**a**,**b**) QTL for grain protein and groat oil content in the Ogle x Marion population are closely linked on one linkage group and share a common marker on another.; (**c**) DArT markers (highlighted) map the single protein content QTL in Dal x Exeter.

In 2004, using AFLP and RFLP, QTL analysis for protein content in groats mapped three QTL in the Terra x Marion population (GrainGenes Mapdata: Oat-2004-TxM) [6], two of which had significant markers that were genetically mapped at the time. One of the grain protein QTL, similar to the one for test weight, overlaps with the N1 locus for the covered/hulless trait (Figure 2a). That same year, 17 QTL were described in an Ogle x MAM17-5 population (GrainGenes Mapdata: Oat-2004-OxM-QTL4) for protein content and five QTL were reported for oil content [7]. Several QTL are linked in the population (Figure 3a) and one RFLP marker in the OxM population was significant for both oil and protein content QTL (Figure 3b). In 2012, a Dal x Exeter population was mapped with DArT markers (Diversity Arrays Technology, Pty Ltd., Canberra, Australia) primarily mapping QTL for total oil and content of individual fatty acids (see below). Analysis of protein content was included in that study, and a single QTL for protein content with two linked significant markers was mapped (Figure 3c) (GrainGenes Mapdata: Oat-2012-Dax_x_Exeter) [31]. Protein content was measured again in 2014 in the Iltis advanced backcross populations and only one QTL in each population was discovered and aligned on the linkage groups among AFLP markers (Figure 2b) [15].

### 3.4. Fat Content

Similar to protein, fats are a key part of the diet. A high fat content is important for animal feed, providing metabolizable energy. In contrast, high fat in the form of groat oil in oat products is generally not desirable for human consumption, because it may hasten rancidity and shorten the storage life [5]. Grain fat content for oat is parsed into six separate traits in GrainGenes: (total) groat oil, linoleic acid content, linolenic acid content, oleic acid content, palmitic acid content, and stearic acid content. Oleic acid is a monounsaturated fatty acid, linolenic and linoleic are polyunsaturated fatty acids, and stearic and palmitic are saturated fatty acids.

**Figure 4:**
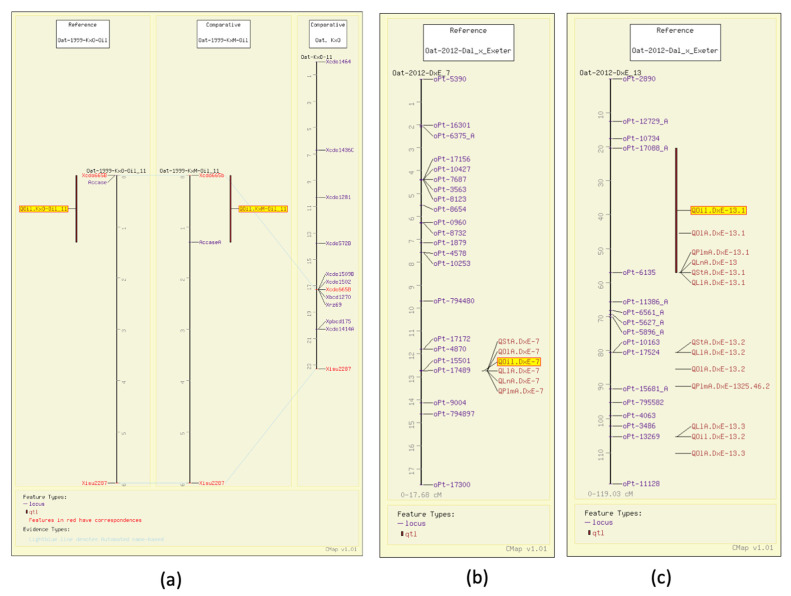
Groat oil content QTL illustrated on the CMap map viewing/comparative mapping tool. (**a**) Total groat oil content (highlighted) in Kanota x Marion and Kanota x Ogle populations is linked to ACCase. By aligning a consensus map published later, the region around the gene of interest can be populated with additional markers. (**b**,**c**) Total groat oil QTL (highlighted) mapping with the QTL for content of the five individual fatty acids.

Early on, Kianian et al. determined groat oil content to be linked to an enzyme responsible for the first committed step of de novo fatty acid biosynthesis, acetyl-CoA carboxylase (ACCase) [32]. They mapped that gene with aligned QTL for groat oil content, along with common markers in Kanota x Ogle and Kanota x Marion (GrainGenes Mapdata: Oat-1999-KxO-Oil and Oat-1999-KxM-Oil). Figure 4a illustrates these linkage groups and the ability of the comparative mapping tool, CMap, to populate the number of molecular markers around genes of interest. This allows breeders to track ‘favorable’ QTL with marker-assisted selection (MAS) using genetically linked markers early on in the breeding process.

Additional QTL have been mapped for groat oil content, including five mapped in Terra x Marion (GrainGenes Mapdata: Oat-2004-TxM), with one associated with the N1 locus for the covered/hulless trait (Figure 2a) [6]. Six groat oil content QTL were mapped in Ogle x Marion, and as mentioned above, one aligns with a QTL for protein content (GrainGenes Mapdata: Oat-OxM-2004-QTL4) (Figure 3b) [7]. One QTL in each of the Iltis advanced backcross populations align on CMap (Figure 2b) [15].

As described above, in a study on a well-populated DArT map for Dal (high oil) x Exeter (low oil) (GrainGenes Mapdata: Oat-2012-Dax_x_Exeter), Hizbai et al. [31] set out to map QTL for total groat oil, and the content of the fatty acids linoleic acid (18:2), linolenic acid (18:3), oleic acid (18:1), palmitic acid (16:0), stearic acid (18:0), as well as total protein. They also mapped agronomic QTL for plant height, heading date, and lodging. Of the six groat oil content QTL, two were associated with all individual fatty acid content QTL (Figure 4b,c). Total QTL mapped for individual fatty acid QTL content, including those that co-mapped with total oil, were six linoleic acid, four linolenic acid, four palmitic acid, eight oleic acid, and four stearic acid content QTL, several of which share significant markers. 

### 3.5. Beta-Glucan Content

Oat ‘beta-glucans’ or ‘(1→3), (1→4)-β-d-glucans’ are a source of soluble dietary fiber gaining worldwide attention for their coronary health benefits [33]. Having a high β-glucan and low fat content in certain oat products are part of the requirement for health claims [5]. As oat breeders are breeding for any of the milling traits discussed so far and more we have not discussed, mapping the QTL for β-glucan content and developing reliable molecular markers will allow breeders to use marker-assisted selection (MAS) during the breeding process. MAS will enable breeders to keep high β-glucan alleles in the lines they develop by testing the DNA in their breeding program early on for the favorable markers. 

Early genetic mapping projects to find QTL in the bi-parental populations described above, often tested the lines for β-glucan (BG) content in the grain, assuming parents differed for that trait. Kianian et al. found seven QTL in Kanota x Ogle and four QTL in Kanota x Marion populations (GrainGenes Mapdata: Oat-2001-KxO-QTL and Oat-2001-KxM-QTL [34]. De Koeyer et al. mapped five QTL in the Terra x Marion population (GrainGenes Mapdata: Oat-2004-TxM) [6]. Hermann et al. mapped three QTL in the Iltis advanced backcross populations (two in ‘A’ and one in ‘B’; GrainGenes Mapdata: Oat-2014-AB_QTL_PopnA, Oat-2014-AB_QTL_PopnB) (Figure 2b) [15]. 

In 2020, two large genome-wide association studies (GWAS) were published that identified β-glucan content QTL in association mapping panels using single nucleotide polymorphism (SNP) markers. Seven QTL were mapped from a genetically diverse inbred line collection of 431 genotypes, most of which were developed by the Federal University of Rio Grande do Sul (UFRGS) Oat Breeding Program in Brazil [9]. Fogarty et al. [10] identified 58 significant markers from three panels of elite accessions (CORE) and confirmed the role of cellulose synthase-like F6 (AsClsF6) genes in β-glucan biosynthesis in oat. AsClsF6 clones from the A, C, and D genomes in hexaploid oat from multiple cultivars of *A. sativa* and diploid species were sequenced and submitted to GenBank. A SCAR marker for the ASCslF6 locus on chromosome 7D was developed and mapped in a HiFi x CDC Sol-Fi bi-parental population [10].

## 4. Oat Genome Browser Views 

Genome browsers integrate a genome sequence with annotations, enabling researchers to visualize, query, download, and analyze data within a genomic context. GrainGenes uses the JBrowse application [35] and hosts 55 individual genome browsers, four of which are for oat. Two are for the diploid oats, *Avena atlantica* and *Avena eriantha* [4], and two are versions of the PepsiCo OT3098 hexaploid map from 2020 and 2021 (https://wheat.pw.usda.gov/jb?data=/ggds/oat-ot3098v2-pepsico, accessed on 17 March 2022). In addition to the pseudomolecules (i.e., the DNA sequences spanning whole chromosomes), the browser displays the locations of genotyping-by-sequencing (GBS) markers and G-quadruplexes that are implied in transcription, replication, and recombination [36,37,38]. The quadruplex datasets are available for download through each genome browser track’s “Save this track” menu option, which appears when the track name is clicked in the browser section. They are also available in the Supplementary Materials sections of the papers cited above. GrainGenes personnel have created a detailed YouTube video tutorial about how to access genome browsers and download data called “Saving Information from GrainGenes Genome Browsers”, which is available at https://wheat.pw.usda.gov/GG3/tutorials, accessed on 17 March 2022.

**Figure 5:**
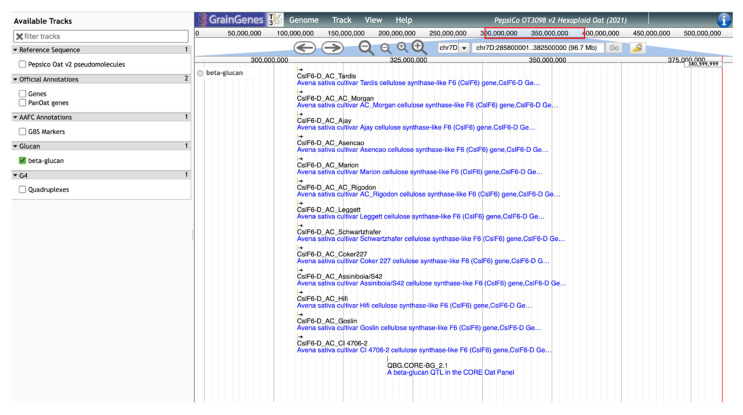
JBrowse view of the manually curated track for β-glucan QTL and cloned alleles for cellulose synthase-like (Csl) aligned on the 7D chromosome of the PepsiCo OT3098 v2 browser.

GrainGenes’ additional contributions to the content of the OT3098 oat browser are manually curated tracks layering genetically derived information into a physical space. As an example, significant SNP markers from the β-glucan QTL identified by Zimmer et al. [9] and Fogarty et al. [10] were located on the OT3098 v2 genome using the BLAST [16,17] tool available in GrainGenes (https://wheat.pw.usda.gov/blast/, accessed on 17 March 2022). The track includes the positions of cellulose synthase-like (Csl) AsCslF6 alleles identified by Fogarty et al. [10]. The positions of the matches determined from the BLAST results were used to produce a new, curated, quality track on the browser displaying GWAS results, QTL, and cloned AsClF6 alleles from 13 cultivars (Figure 5).

**Figure 6:**
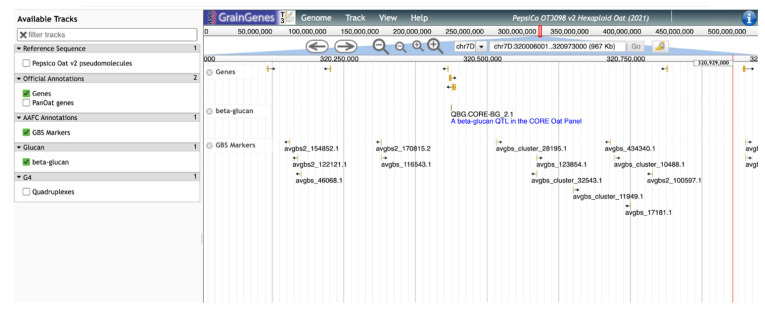
A ‘zoomed-in’ view of the QTL from Figure 5. Additional tracks show annotated genes and SNP markers.

This β-glucan track provides a sequence-level visualization of the locations of genes and markers related to β-glucan in oat and will allow breeders and others to select the most appropriate markers for further selection and characterization of germplasm for high β-glucan lines (Figure 6). QTL tracks will also assist researchers looking to determine which genes and alleles are most important for this trait in certain genetic backgrounds and under different environmental conditions, once gene expression data are included in the annotations. As the GrainGenes database accumulates more information over time, tracks summarizing information concerning other oat quality traits will be continuously added.

## 5. Reaching out to the GrainGenes Team

The GrainGenes team is always happy to assist users. If users cannot access information easily or would like to have assistance with our tools or pages, they can use the “Feedback” link at the top of every GrainGenes page to reach the GrainGenes Team. 

## Figures and Tables

**Table 1:** Gene Classes with number of *Avena* genes in parenthesis in GrainGenes.

Acetyl Co-A Carboxylase (1)	Ligule Development (4)
Acid phosphatase (2)	Malate dehydrogenase (2)
alpha-Amylase (2)	Male sterility (2)
alpha-Esterase activity (2)	Maturity (1)
Avenin (7)	Multiple glumes (1)
Awn pubescence (1)	NADH dehydrogenase (1)
Awnedness (6)	Naked karyopsis (4)
Basal articulation (9)	Nodal pubescence (2)
beta-Esterase activity (1)	Nonheading (1)
beta-Galactosidase (1)	Panicle type (7)
beta-glucanase (1)	Pathogenesis-related protein (1)
beta-n-Acetylglucosaminidase (1)	Peduncle development (1)
Blast (1)	Peduncle length (1)
Blunt spikelet (1)	Peroxidase (7)
Chlorophyll deficiency-albino (6)	Phosphatase (1)
Chlorophyll deficiency—albovirescens (1)	Phosphoglucomutase (1)
Chlorophyll deficiency—chlorina (4)	Phosphogluconate dehydrogenase (2)
Chlorophyll deficiency—lutescens (3)	PHYA (1)
Chlorophyll deficiency—netting (2)	Plumule color (1)
Chlorophyll deficiency—stripe (3)	Rachilla pubescence (5)
Coleoptile color (1)	Reaction to *Ditylenchus dipsaci* (1)
Daylength insensitivity (1)	Reaction to *Erysiphe graminis* DC. (5)
Diaphorase activity (1)	Reaction to *Helminthosporium victoriae* (1)
Dwarf (1)	Reaction to *Heterodera avenae* (3)
Dwarfness (9)	Reaction to *Pseudomonas coronafaciens* (3)
Esterase (13)	Reaction to *Puccinia coronata* f. sp. *avenae* (119)
Fatuoid (1)	Reaction to *Puccinia graminis* Pers. (17)
Floret development (1)	Reaction to *Puccinia recondita* Rob. ex Desm. (1)
Floret disjunction (2)	Reaction to *Schizaphis graminum* Rond. (1)
Gametophyte (1)	Reaction to *Ustilago avenae* (11)
Giantism (2)	Reaction to *Ustilago avenae* and *Ustilago kolleri* (9)
Growth habit (1)	Reaction to *Ustilago kolleri* (10)
Isocitrate dehydrogenase (1)	Red seedling pigmentation (3)
Kernel pubescence (11)	Response to vernalization (1)
Leaf blade pubescence (2)	Seed pigmentation (1)
Leaf margin pubescence (2)	Seed proteins (1)
Leaf necrosis (1)	Semiglumeless (1)
Leaf sheath pubescence (4)	Shikimate dehydrogenase (1)
Lemma color (16)	Straw color (1)
Lemma fluorescence (2)	Synapsis (5)
Lemma pubescence (11)	Tertiary seedset (2)
Lemma waxiness (1)	Time to flowering (1)
Lethal (4)	Vivipary (3)
Leucine aminopeptidase activity (1)	

**Table 2:** Trait Categories with number of *Avena* QTL in GrainGenes in parenthesis.

Awn Frequency (2)	Milling Difficulty (oat) (1)
beta-glucan (FIA) (13)	Milling yield (oat) (2)
beta-glucan (NIR) (3)	Naked (2)
beta-glucan (oat) (88)	Oat winter hardiness (crown meristem freeze tolerance) (1)
Biomass (3)	Oat winter hardiness (field survival) (1)
BYDV (58)	Oleic acid content (8)
Days to heading (136)	Palmitic acid content (4)
Days to maturity (2)	Panicle number (2)
Flowering time (56)	Panicle type (5)
Grain filling period (2)	Phenology (1)
Grain yield (oat) (15)	Physiological maturity (2)
Groat oil (29)	Protein (1)
Groat percentage (46)	Reaction to barley yellow dwarf virus (2)
Groat protein (24)	Reaction to crown rust (oat, adult) (62)
Growth phase index (3)	Reaction to crown rust (oat, seedling) (11)
Harvest index (1)	Reaction to powdery mildew (8)
Height (155)	Root growth rate (30)
Kernel area (oat) (5)	Root surface area (6)
Kernel length (8)	Severity of crown rust (oat, adult) (56)
Kernel percent hull (3)	Shoot growth rate (14)
Kernel plumpness (4)	Shoot length (3)
Kernel weight (20)	Stearic acid content (4)
Kernel width (oat) (5)	Straw stiffness index (6)
Kernels per spikelet (1)	Straw yield (23)
Lemma pigment (2)	Test weight (63)
Linoleic acid content (7)	Thin kernels (5)
Linolenic acid content (4)	Tillers per plot (9)
Lodging (9)	Visual score (1)
Lodging at flowering (2)	Yield (79)
Lodging at harvest (4)	Aluminum tolerance (*A. strigosa*) (4)

**Table 3:** Ten most recent *Avena* genetic map sets in GrainGenes available for using the CMap tool for comparative mapping.

Map Name	Date	Parent	Markers	Type	Maps
Oat-2020-CORE-SV	2020	Oat Core Collection	80	53 Seed Vigor QTL, SNP, SSR	22 linkage groups
Oat-2020-UFRGS-Bg	2020	UFRGS Panel	27	27 b-glucan QTL, SNP, STS	6 linkage groups
Oat-2020-UMNF-Pc	2020	UMN Founder Population	42	43 Pc, HD QTL, SNP, RFLP	7 linkage groups
Oat-2020-Celx9210_Pc39	2020	Celer x STH9210	66	DArT, SNP, SCAR, Pc39	1 linkage group
Oat-2020-CORE-BG	2020	Oat Core Collection	219	56 QTL, SNPs	19 linkage groups
Oat-2020-HxS-BG	2020	HiFi x Sol-Fi	4	AsCslF6_D, SNPs	1 linkage group
Oat-2020-Pc98	2020	Pc98, Bingo, Kasztan	21	KASPs, Pc98	Mrg20 in 2 popns
Oat-2019-TX07CSxHidalgo	2019	TX07CS-1948 x Hidalgo	6721	SNPs, 31 QTL for seedling crown rust, height, heading date	21 Linkage Groups
Oat-2019-CrownRust	2019	9 parents (see mapdata)	36	SNPs Pc45, PcKM	1 Linkage group from 5 popns
Oat-2018-Consensus	2018	Oat Diversity Panel	99,846	SNP, RFLP, Genes	21 Merged Groups

## Data Availability

All data discussed and illustration in this communication are available at the GrainGenes database at http://wheat.pw.usda.gov, accessed on 17 March 2022.
